# Proteoliposomes as Tool for Assaying Membrane Transporter Functions and Interactions with Xenobiotics

**DOI:** 10.3390/pharmaceutics5030472

**Published:** 2013-09-18

**Authors:** Mariafrancesca Scalise, Lorena Pochini, Nicola Giangregorio, Annamaria Tonazzi, Cesare Indiveri

**Affiliations:** 1 Laboratory of Biochemistry and Molecular Biotechnology, Department BEST (Biology, Ecology and Earth Sciences), University of Calabria, Via P. Bucci 4c, Arcavacata di Rende 87036, Italy; E-Mails: mscalise@unical.it (M.S.); lorena.pochini@unical.it (L.P.); 2 CNR Institute of Biomembranes and Bioenergetics, via Amendola 165/A, Bari 70126, Italy; E-Mails: n.giangregorio@ibbe.cnr.it (N.G.); a.tonazzi@ibbe.cnr.it (A.T.)

**Keywords:** proteoliposomes, transport, drugs, xenobiotics, reconstitution, drug discovery

## Abstract

Proteoliposomes represent a suitable and up to date tool for studying membrane transporters which physiologically mediate absorption, excretion, trafficking and reabsorption of nutrients and metabolites. Using recently developed reconstitution strategies, transporters can be inserted in artificial bilayers with the same orientation as in the cell membranes and in the absence of other interfering molecular systems. These methodologies are very suitable for studying kinetic parameters and molecular mechanisms. After the first applications on mitochondrial transporters, in the last decade, proteoliposomes obtained with optimized methodologies have been used for studying plasma membrane transporters and defining their functional and kinetic properties and structure/function relationships. A lot of information has been obtained which has clarified and completed the knowledge on several transporters among which the OCTN sub-family members, transporters for neutral amino acid, B0AT1 and ASCT2, and others. Transporters can mediate absorption of substrate-like derivatives or drugs, improving their bioavailability or can interact with these compounds or other xenobiotics, leading to side/toxic effects. Therefore, proteoliposomes have recently been used for studying the interaction of some plasma membrane and mitochondrial transporters with toxic compounds, such as mercurials, H_2_O_2_ and some drugs. Several mechanisms have been defined and in some cases the amino acid residues responsible for the interaction have been identified. The data obtained indicate proteoliposomes as a novel and potentially important tool in drug discovery.

## 1. Introduction

Membrane transport systems play pivotal roles in cell homeostasis. This is indirectly but definitely demonstrated by the occurrence of many human pathologies of various severity caused by defects of genes coding for transporters [1,2,3,4,5].

The functions of transport systems are at the basis of metabolite exchanges among cells and extracellular environment and, within a cell, among different sub-cellular compartments, allowing completion of compartmentalized biochemical pathways. It was previously believed that a large number of molecules could diffuse through membranes, but it is now established that transmembrane proteins are necessary for translocating virtually all the molecules with the exception of very few small compounds such as oxygen [6,7]. Transport systems are hydrophobic transmembrane proteins which can be classified using different criteria based on functional, molecular [8] or evolutionary [9] aspects.

**Figure 1:**
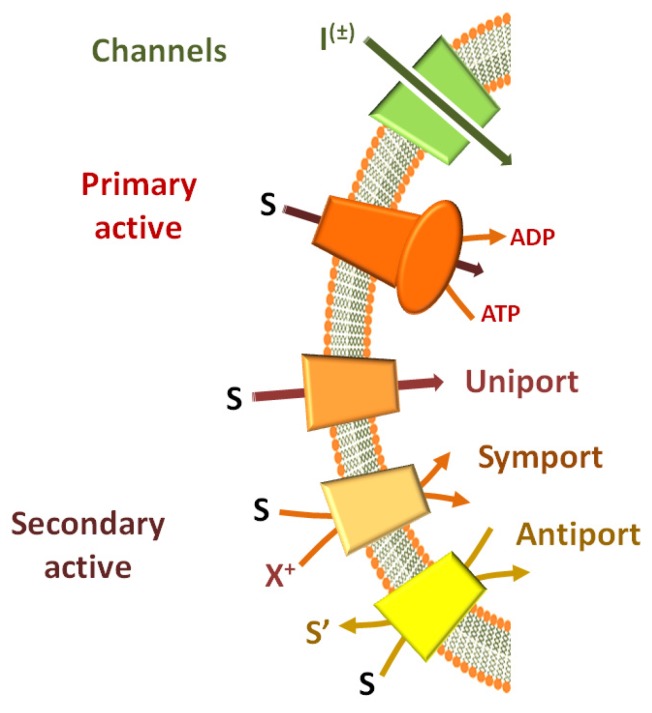
Functional classification of membrane proteins. Channels are depicted in green and I^(±)^ represents the translocated ion. S represents the transported substrate in both primary and secondary active transporters. ATP hydrolysis for primary active transporters is highlighted. Transport mechanisms of secondary active transporters are described (uniport, symport and antiport). In the symport scheme, X^+^ indicates a generic cation and its concentration difference drives the transport. In the antiport scheme, S’ indicates the countersubstrate driving the vectorial reaction.

A well established functional classification [10], which fits also some molecular aspects, groups transport systems in two main categories: channels and permeases (Figure 1). The first group is constituted by transmembrane proteins which mainly catalyze ion transport with turnover rates normally much higher than 1000 min^−1^. The driving force for transport derives from ion concentration gradients. The high transport rate of ions generates measurable currents which can be detected by electrophysiological methods. Important exceptions are aquaporins which do not generate currents [11]. Permeases catalyze transport of a huge number of different compounds with a turnover much lower than channels. Therefore, even in the case of ionic substrates, permeases normally do not generate currents of sufficient intensity to be measured by electrical devices. Thus, different methodologies have to be employed for their study. The most common one is the use of radioisotope labeled substrates. On the basis of the origin of the transport driving force, permeases are subdivided into primary and secondary active transporters (Figure 1). Primary active transporters contain ATPase domains (ABC, *i.e.*, ATP Binding Cassette) or subunits which generate free energy from ATP hydrolysis coupled to the transport process. These transporters are also called “pumps” for their ability to drive transport of substrates against their concentration gradient. Secondary active transporters which constitute the largest group, can be classified in uniporters, symporters and antiporters. The driving force for this group of transporters is generated, respectively, by concentration gradients of the transported substrates, coupling to a co-transported ion such as Na^+^ or H^+^, or coupling to a countersubstrate which is transported in the opposite direction. In the case of ion-coupled transport, Δψ or ΔpH can also contribute to the driving force. For the reasons described above, the secondary active transporters are responsible for catalyzing the transmembrane translocation of most of the compounds which are necessary for the metabolic cell demands. In humans, indeed, many secondary transporters are involved in absorption of glucose [12] and other monomeric carbohydrates [13], amino acids [14,15,16], lipids [17] and a large variety of cofactors which are essential for metabolic purposes [18,19,20]. Then, transporters mediate the trafficking of specific metabolites through intracellular membranes of mitochondria [21], endoplasmic reticulum [22], or intracellular vesicles [23,24], excretion of catabolites and reabsorption of ions [25,26] and many endogenous compounds [27,28] at the kidney level.

The existence of a very large number of different transport proteins has been confirmed by genome wide sequencing which revealed that genes coding for these proteins are much more numerous than earlier predicted. More than 400 genes coding for secondary active transporters have been annotated in *H. sapiens* [10,29] so far. In addition, channels and ABC transporters are required for neurotransmission, maintenance of electrochemical ion gradients, active extrusion of xenobiotics. The number of mature transport proteins in the different tissues and cells may be much higher than coding genes, due to alternative splicing events (see Section 3). Secondary active transporters have been classified as SLC (SoLute Carriers) with additional alphanumerical codes indicating the sub-family and the specific transporter. Many of these genes have been cloned and the gene products have been identified in human or animal tissues and functionally characterized. No structure of human secondary active transporter has been resolved so far [8,30,31]. In the recent years it has been widely shown that many of these transporters are involved in interactions with xenobiotics among which drugs and toxic compounds, such as heavy metals [32,33,34,35,36,37,38,39]. In this respect, transporters can be involved in absorption and hence in drug delivery or, upon off-target interactions [40], transporters can also be responsible for side effects of drugs [41,42,43]. In addition to the described physiological and pathophysiological roles, it has been revealed that the expression of many transport systems is altered in cancer cells to accomplish their specific metabolic needs. These findings have important outcomes in human health since the altered proteins represent potential pharmacological targets [36,44,45,46,47,48,49]. For all these reasons, the International Transporter Consortium compiled a series of recommendations and draft guidance documents on interactions of transporters with drugs [39]. The challenges of transporter studies in designing new pharmacological compounds and in testing safety of widely diffused drugs and other xenobiotics have been recently highlighted [50,51]. Among the recommendations, a major task consists in the development of tools for studying the function of transport systems and defining their interaction with xenobiotics. FDA recently recommended considering membrane transporters in drug design and identification of side effect targets. In particular, FDA recommends identification of the principal routes of elimination, quantifying the contribution to drug disposition not only by enzymes but also by transporters which can have important effects on pharmacokinetics and drug exposure. Indeed, differently from enzymes, which are largely concentrated in the liver and intestine, transporters are present with varying abundance in all tissues playing, therefore, more general roles in drug absorption, distribution and elimination [52,53,54].

## 2. Experimental Tools for Studying Transport

Due to their hydrophobic nature, the physiological and biochemical knowledge of transport proteins had a large delay with respect to that of soluble enzymes. After 1970, some pioneer studies on transporters started.

### 2.1. Intact Cell Systems

To assay transport functions, the flux of labeled compounds through native membranes was followed in cell systems [55] or isolated organelles, such as mitochondria [56] or microsomes [57], derived from endoplasmic reticulum. Intact cell models are still widely used as tools for studying the properties of transport systems. A section of the FDA Guidance related to “*In Vitro* Transporter Studies”, proposes the use of Caco-2 cells or cell lines over-expressing specific transporters, as the preferred method for evaluating drug-transporter interactions before experimentation in animals or humans [39,58]. The most common systems are intact cells expressing endogenous transporters or specific cell lines over-expressing homologous or heterologous transporters. The first one has the advantage of containing native functional transporters and also interactors or accessory proteins which, in some cases, contribute to or regulate their function. The transient or stable over-expressing systems, such as *X. laevis* oocytes or tumor cell lines have, on the other hand, the advantage of increasing the amount of the protein of interest. In such a way the activity of the expressed protein overcomes that of the endogenous ones, leading to a better resolution of the transport activity. The described intact cell experimental systems allowed identification and functional classification of a large number of transport systems from plasma and intracellular membranes. However, the complexity of both experimental models hampers the characterization of the transporters at the kinetic and molecular levels and gives rise to several interferences caused by the presence of many similar or different transporters in the same membrane and by the intracellular enzyme pathways that can readily metabolize the substrates used for measuring the transport activity. Moreover intact cells do not allow free access to the intracellular compartment and, hence, to the internal site of the transport protein. The use of inhibitors is essential to discriminate the transport catalyzed by a specific transporter from that catalyzed by other similar transporters in intact cells. However, it can never be excluded that inhibitors may interact with the other transporters, as well. This, for example occurs in the case of amino acid transporters, which are redundant in mammalian cells and show overlapping specificities for several amino acids and inhibitors. All these interferences hamper the study of transport kinetics. Therefore, due to the described limitations, many aspects of the structure, function and regulation of the transporters are still controversial or unknown.

### 2.2. Proteoliposomes

To improve the knowledge of transporters, alternative strategies for assaying transport have been developed since the beginning. But, only more recently these strategies have been implemented and optimized following the evolution of methodologies of handling hydrophobic proteins. This led to an exponential increase of the studies on transporters with a huge number of published papers. In the last decade, suitable heterologous expression systems for membrane proteins have been pointed out and several transporters have been produced and purified in large scale [21,30,59]. These pure proteins are suitable for both functional and structural studies. However, no structures of human transporters are, so far, available except those of some channels and ABC transporters [60,61,62,63]. On the contrary, the functional studies have been better implemented using proteoliposomes as an experimental model to define function, kinetics and regulation of purified transporters.

Liposomes were initially produced for encapsulating and delivering enzymes [64] or pharmaceutical compounds [65,66,67]. Liposomes with transport proteins embedded into the membrane bilayer (proteoliposomes) have been then used as a tool for studying membrane transporters in an isolated environment [68,69,70].

#### Methods of Proteoliposome Preparation

**Figure 2:**
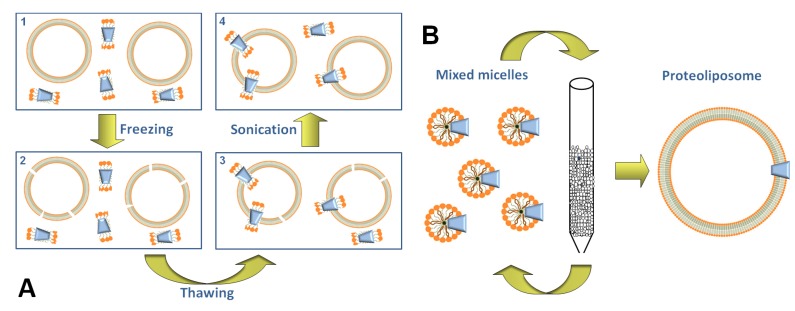
Sketch of methodologies for proteoliposome preparation. (**A**) the four steps of freeze-thaw sonication: (**1**) mixture of preformed liposome with protein and detergent; (**2**) freezing of the mixture in liquid N_2_ with consequent rupture of vesicle membranes; (**3**) slow thawing at 0 °C causing the insertion of the protein, solubilized in detergent, into the membrane; (**4**) mild sonication step (low energy in pulse mode) which facilitates the sealing of proteoliposomes; (**B**) cyclic chromatography on column: mixed micelles of detergent, phospholipids and protein are shown; detergents and phospholipids are represented by an orange hydrophilic head and one or two carbon chains, respectively. The reconstitution mixture is passed through a column containing hydrophobic resin (Bio-Beads), for 10–20 times. This led to detergent removal with formation of liposomes containing the protein into the membrane bilayer (proteoliposomes).

One of the first methods for preparing proteoliposomes was based on sonication of phospholipid mixtures with transport proteins extracted from biological samples [68,70]. Soon after, the freeze-thaw-sonication procedure was pointed out, which consists of freezing mixtures containing liposomes and transport proteins solubilized in non-ionic detergents and then in slowly thawing these samples (Figure 2A). After freezing, liposomes are broken due to ice formation and, during the slow thawing, the proteins insert into the phospholipid bilayer. Mild sonication of the formed proteoliposomes reduces the leakage of the vesicles and the presence of multilamellar structures [69,71]. This procedure has been used for long time and over the years it has been optimized for mitochondrial transporters [72]. The main disadvantage of this methodology for proteoliposome preparation is the presence of detergent, even though in small amounts, which is necessary for solubilizing the hydrophobic transport proteins. The residual detergent can cause leakage of the membrane and the insertion of the transport proteins in the phospholipid bilayer in a random orientation, *i.e.*, not reproducing the orientation in the native cell membrane.

A more suitable methodology for liposome formation consists in starting from a mixture of protein, detergent and phospholipids from which the detergent is removed by hydrophobic resins or, in few cases, by dialysis. Two main procedures have been conceived for removing the detergent using hydrophobic resins: the cyclic chromatography on column and the batch-wise procedure. The first was conceived by Ueno *et al.* in 1984 [73], then modified [74] for reconstituting the ADP/ATP and glutamate/aspartate mitochondrial transporters and later optimized for other mitochondrial transporters [75]. This methodology is based on repeated chromatography of the reconstitution mixture on the same hydrophobic column for 10–20 cycles, up to the nearly complete removal of the detergent (Figure 2B). In the case of the batch-wise procedure the mixture of protein, detergent and phospholipids is incubated with the hydrophobic resin, instead of passing through column, for a time sufficient for removing virtually all the detergent [76,77,78]. An important improvement in the detergent removal procedure with respect to the freeze-thaw-sonication is given by the absence of detergents. Under this condition the proteoliposomes are more sealed, unilamellar and, hence, virtually impermeable to hydrophilic compounds. An additional very important outcome of the detergent removal method consists in the insertion of the protein in an unidirectional orientation, which in most cases, luckily corresponds to that of the transporter in the native membrane [79], *i.e.*, the extracellular side of the transporter is outwardly oriented also in the artificial membrane (right-side-out orientation). This is due to the feature of the starting micelles which have a relatively small radius, *i.e.*, of the same order of magnitude of the protein size. This property forces the asymmetric protein, to insert into the micelles with the tight side inwardly directed. After removal of the detergent, virtually all the protein molecules will remain into the phospholipid bilayer with the same orientation (Figure 2B). The experimental system of proteoliposome, carrying functional proteins rightly inserted into the phospholipid membrane, is a very suitable tool to obtain functional information. A modification of the described procedure was introduced, which, in some cases, led to an improvement of the protein insertion into the phospholipid bilayer [80]. The main variations consisted in preparing unilamellar liposomes by reverse phase evaporation, and destabilizing the liposomes by detergent up to their complete solubilization, before removing the detergent by BioBeads. This strategy gave the additional advantage of monitoring the efficiency of proteoliposome formation.

## 3. Transporter Function in Proteoliposomes

### 3.1. Using the Freeze-Thaw-Sonication Method

After the work by Kasahara and colleague [69] on the reconstitution of the human D-glucose transporter purified from ghost erythrocytes, several pioneering studies were performed on mitochondrial transporters (carriers) which, for their smaller size, are easier to handle than plasma membrane transporters. The mitochondrial ADP/ATP carrier has been characterized clarifying several aspects of the transport, among which the electrogenic nature of the ADP/ATP antiport reaction. The same procedure was also used for assaying the phosphate and citrate carriers, which have been well characterized in this system [81,82,83]. The freeze thaw-sonication procedure, with some modifications and optimization, has been more recently used for studying some human plasma membrane transporters obtained by recombinant expression in various hosts [84,85,86]. In the case of the nucleoside transporter hENT1 the liposome reconstitution combined with site directed mutagenesis, allowed to establish that *N*-glycosylation is not essential for transport function [84]. Important information were also derived from reconstitution studies on the function of the human sodium dependent glucose transporter, hSGLT1; the calculated kinetic parameters mirrored, indeed, those measured in cell systems confirming the suitability of proteoliposomes. In this system it was possible to assay the effect of cholesterol that could not be studied in intact cells. It was found that the lipid stimulated glucose transport, but was not essential for the activity of hSGLT1. Also in this case it was assessed that *N*-glycosylation does not play any role in the transport function but, probably, in the trafficking of the transport protein towards the plasma membrane [85]. These findings correlated well with a similar conclusion drawn by different experimental approaches for another plasma membrane transporter, the OCTN2 [87]. hMATE2, the isoform 2 of the human transporter for Multidrug And Toxic compound Extrusion was also studied using proteoliposomes obtained by a modification of the freeze-thaw procedure, adding a dilution step. This method allowed the specific characterization of this transporter together with the shorter splice variant hMATE2K showing that in renal tubules these proteins are involved in pH dependent organic cation transport [86]. An important example of liposome reconstitution by freeze-thaw-sonication procedure is represented by three members of SLC22 family, the organic cation transporters OCT1 and OCT2 and the anion transporter OAT1. These transporters are involved in elimination and distribution of drugs, toxins end endogenous compounds. The rat isoforms of these transporters were over-expressed in an *E. coli* derived cell-free system, then purified and reconstituted in liposomes. Using this methodology, the Km values of OCT1 and OCT2 for their substrate, 1-methyl-4-phenylpyridinium, have been assessed. The inhibition by many organic cations has been described as well as the electrogenicity of transport and the substantial irrelevance of *N*-glycosylation. On the other hand it has been shown that OAT transports *p*-aminohippuric acid by electroneutral exchange with ketoglutaric acid [88].

### 3.2. Using the Detergent-Removal Method

After its development, this method has been widely used, since it allows more reproducible experimental conditions, as above described. Several mitochondrial transporters were reconstituted and functionally studied as extensively reviewed [21,75,83]. The methodology of detergent removal, after appropriate modification, was then applied to plasma membrane transporters. These transport proteins are larger than the mitochondrial ones and contain in most cases, hydrophilic strands of more than one hundred amino acids, which hamper their refolding. In the last years some successful reconstitutions of rat plasma membrane transporters extracted from animal tissues were obtained [89,90]. The carnitine transporter of plasma membrane (OCTN2) extracted from rat kidney was firstly reconstituted in proteoliposomes [89]. The pointed out tool allowed the characterization of function and kinetics of the transporter which have been recently reviewed [30,59]. This transporter mediates a Na^+^-dependent antiport of carnitine with itself or with carnitine derivatives, which represents an exception to the standard classification (Figure 1), since an antiport is coupled to a symport mechanism. Soon after, some amino acid transporters were investigated using proteoliposomes. The neutral amino acid transporters ASCT2 and B0AT1 were firstly extracted from rat kidney and reconstituted in proteoliposomes. Some functional properties in proteoliposomes, which were found to be very similar to those previously described in intact cells, indicated the correspondence of the reconstituted transporter with the ASCT2 and B0AT1 transporters identified in cell systems [90,91]. However, due to the unique features of the proteoliposome tool, novel functional properties were revealed. ASCT2, which was originally named for its ability to transport Alanine, Serine, Cysteine, was shown to transport also glutamine, besides other neutral amino acids by a Na^+^-dependent antiport mode of transport, which represents a further exception to the classification of Figure 1. Glutamine was shown to be transported bi-directionally in line with the specific localization of ASCT2 in the renal epithelium where it may regulate glutamine and other neutral amino acids reabsorption. The use of proteoliposomes, moreover, shed new light on the regulation of transport mediated by ASCT2 revealing activation by ATP and the importance of some Cys specifically targeted by thiol reagents [90]. The mechanism of the unusual ter-reactant reaction taking place among internal glutamine, external glutamine and external Na^+^ was investigated. In this kind of experiments a pseudo-bi-reactant analysis was performed varying the concentrations of two of the three substrates, keeping constant the third one. After this complex kinetic analysis, the transport mediated by ASCT2 resulted to follow a random simultaneous mechanism, implying the formation of a ternary complex without preferential order for substrates binding and transport. In this scenario the transporter might have one internal and two external sites for substrates [92]. This aspect has been further exploited in order to evaluate specific xenobiotic-transporter interactions (see Section 4).

The B0AT1 transporter extracted from rat kidney was characterized in proteoliposomes. Differently from ASCT2 this transport system catalyzes a Na^+^-glutamine co-transport (symport). Also in this case, novel functional features were highlighted by the proteoliposome studies. The transporter is stimulated by intraliposomal (corresponding to intracellular) K^+^, which is not transported but acts as a modulator. It was also demonstrated that the transporter is strictly dependent on membrane potential, artificially generated in proteoliposomes as K^+^ diffusion potential in the presence of the ionophore valinomycin. In analogy with the ASCT2 transporter, also B0AT1 was reactive towards thiol reagents owing to the presence of exposed Cys residues [91]. Also in the case of B0AT1, experiments aimed to clarify the transport mechanism have been performed. The bi-substrate kinetic analysis conducted in proteoliposomes showed a simultaneous random mechanism [93]. This finding shed definitive light on this aspect clarifying the discrepancies previously arisen from the studies performed in cell systems. Furthermore it has been clarified that collectrin and ACE2, known as modulators of amino acid fluxes, are not involved in the intrinsic transport function of B0AT1 suggesting roles as potential regulators of the transporter expression. An important achievement in the study of membrane transporters is the over-expression and purification, which allow handling of human transporters. An example is the human ammonium transporter RhCG which was reconstituted using a detergent removal procedure based on incubation of the protein/phospholipid/detergent mixture with Bio-Beads upon expression in HEK293 cell lines and purification. The reconstituted system allowed us to clarify that RhCG is an ammonium transporter and that it does not require additional proteins for its function [94]. Large scale production of recombinant mammalian transporters, have been obtained in *E. coli* or *P. pastoris* after optimization of the expression procedures. These strategies allow in some cases the reconstitution of human transporters [78,95,96]. The recombinant Organic Cation Transporter Novel 1 (OCTN1, SLC22A4) has been reconstituted in liposomes. Relationships of the function of this transporter with human pathology have been recently reviewed [59]. The link relies essentially on the capacity of the transporter to mediate efflux of acetylcholine, as revealed in proteoliposomes, which can play an important role in non-nervous tissue [97]. It was further revealed, that the variant hOCTN1-L503F associated to the Crohn’s disease, showed impaired acetylcholine efflux activity [98], opening the way to further inhibition kinetic analysis and structure-function relationship studies with perspectives in drug discovery and design [59]. Another member of OCTN subfamily which has been identified only in mouse, the mOCTN3 (SLC22A21), over-expressed in *E. coli*, has been functionally characterized in proteoliposomes [95]. More recently, the human isoform of the glutamine transporter ASCT2, over-expressed in *P. pastoris* has been reconstituted in liposomes and functionally characterized. Noteworthy, in spite of the high sequence homology between the human and the rat ASCT2 proteins, important differences have been revealed. As an example, hASCT2 is insensitive to ATP. The most interesting finding revealed in proteoliposome is the side-specificity for different amino acids. While glutamine, serine, asparagine and threonine are bi-directionally transported, cysteine, alanine, valine and methionine are specifically inwardly translocated. This functional asymmetry is important for maintaining amino acid homeostasis under physiological conditions. Since this transporter is over-expressed in cancer cells [47], ASCT2 will also represent a potential pharmacological target for cancer therapy (see Section 4). An application of the destabilized liposome strategy [80] has been very recently shown for the MexAB pump from *P. aeruginosa*, able to mediate drug efflux [99]. In this work the efflux pump, a complex of MexA and MexB, has been co-reconstituted in liposomes with bacteriorhodopsin (BR), a light activated pump. The BR, upon illumination, induces the formation of a proton gradient across the proteoliposome membrane which furnishes the driving force for the MexAB drug efflux activity. This working strategy allowed us to explain, at molecular level, the mechanism of drug extrusion, shedding light on the drug resistance described for several pathogens [99].

Modified methodologies for reconstituting transporters were employed in some cases as for the rat GLT-1 glutamate transporter and for the rat vesicular monoamine transporter VMAT2, which were reconstituted using a procedure mainly based on dialysis [100,101]. This approach is relatively simple and low cost, but its use on large scale is hampered by the poor reproducibility, the duration of the experiments (dialysis can last more than 2 days) and the possible retention of molecules on the dialysis membrane [80].

## 4. Using Proteoliposomes for Revealing Xenobiotic-Transporter Interaction Mechanisms

**Figure 3:**
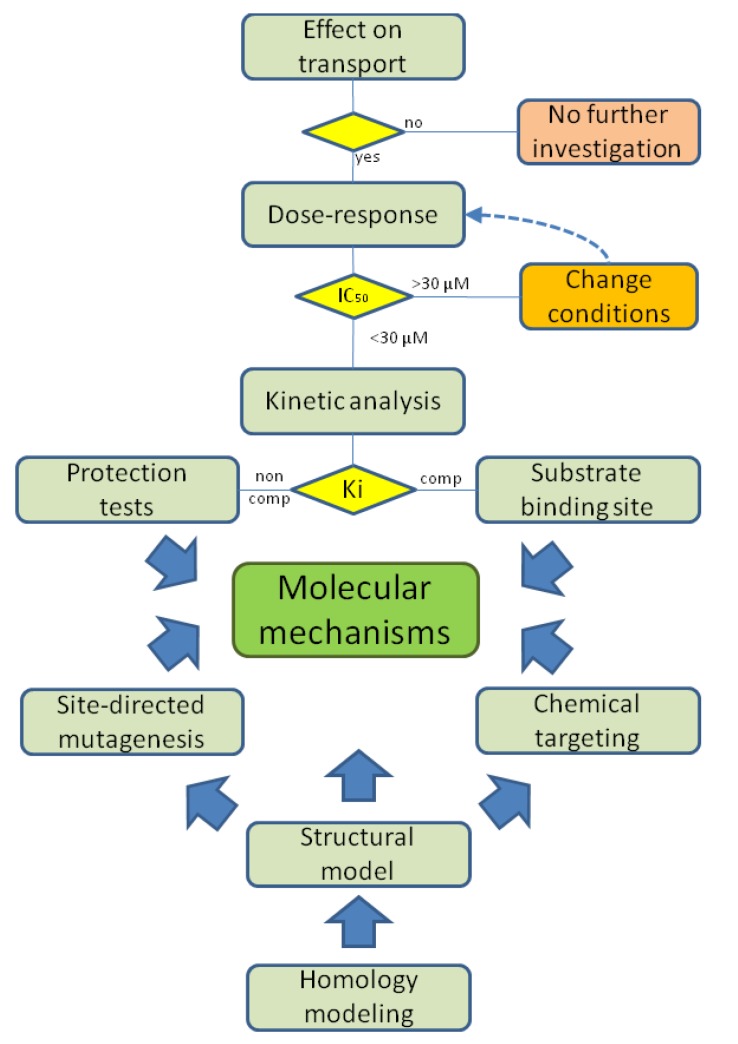
Work plan for xenobiotic-transporter interaction studies in proteoliposomes. When the tested molecule has an effect on transport activity, the dose response experiments are performed with IC_50_ measurement. For those compounds showing an IC_50_ lower than 30 μM the analysis goes forward allowing kinetic parameters evaluation. Inhibition mechanism, competitive (comp) or non competitive (non comp) is deduced and Ki are calculated. Bioinformatics can help in obtaining homology structural models further validated by site-directed mutagenesis (when recombinant protein is available) and/or by chemical targeting of specific amino acid residues. This methodology, together with the inhibition analysis, concurs to the determination of the molecular mechanisms.

The interaction of xenobiotic compounds with a transport system can be considered of interest if the constants, IC_50_ or Ki, are equal or lower than their concentration in plasma or in cells following absorption, or administration in case of drugs. In some instances the maximal IC_50_ threshold for considering the interaction of a compound with a protein target has been fixed at 30 µM or lower [32,40]. However, higher constants may be considered in those circumstances in which pharmacological compounds are administered at much higher doses, as in the case of many antibiotics (see Table 1). When the IC_50_ for the interaction with xenobiotics is sufficiently low, the physiological functions underlined by the transporter, will be altered. This phenomenon can be described in terms of general alterations of transport and of cell functions by studies in intact cell systems. However, for a description of the mechanism of the interaction at the molecular level, proteoliposomes are much more suitable. Indeed, extensive kinetic and molecular analyses of the transporter-xenobiotic interaction can be performed obtaining information on the constants, the type of interaction (competitive, non competitive or mixed inhibition) and, in some cases, the molecular and structural determinants. Information on the involvement of amino acid residues could be obtained if the structure of the transporter is available. For human transporters these types of molecular information can be currently derived only by homology modeling combined with the use of specific chemical labeling. Moreover, when the transporter is available as a recombinant protein, site-directed mutagenesis can be performed to identify the specific amino acid residues responsible for the interaction. A work plan resuming the investigation steps for studying the transporter-xenobiotic interactions is shown in Figure 3. Transporters which interact with exogenous compounds, can be divided into two target groups on the basis of localization in the cell: first level targets, including the transporters localized on the plasma membrane; second level targets, including the transporters localized in subcellular membranes, such as mitochondria or endoplasmic reticulum. In this section recent studies on the interaction of xenobiotic compounds with first and second level targets, performed in proteoliposomes, are reviewed. According to the work plan of Figure 3, toxic compounds such as heavy metal cations or H_2_O_2_ and commonly used drugs or newly synthesized compounds have been tested. Very interestingly, most of the interacting compounds share a common property, *i.e.*, the reactivity towards thiol groups of Cys residues.

**Table 1:** IC_50_ values and half saturation constants of the reconstituted mitochondrial transporters Carnitine/Acyl-Carnitine Transporter (CACT) and Ornithine/Citrulline Transporter (ORCT).

Molecules	CACT		ORCT		References
	IC_50_	Ki	IC_50_	Ki	
HgCl_2_	0.03		1.4		[102,103]
Mersalyl	0.05		1.1		[102,103]
*p*-OHMB			0.59		[102]
*p*-CMBS			0.61		[102]
Cu^2+^			0.71		[104]
Zn^2+^			40		[104]
Cd^2+^			24		[104]
Pb^2+^			50		[104]
Co^2+^			210	94	[104]
Mn^2+^			350	420	[104]
Ni^2+^			83	53	[104]
H_2_O_2_	170	710			[105]
Cefonicid ^(^*^)^	6800	4900			[106]
Cefonicid ^(^**^)^	120				[106]
Ampicillin	7600	9900			[106]
Mildronate	560	530			[107]

IC_50_ and Ki values are reported as µM; ^(^*^)^ covalent interaction ^(^**^)^ non covalent interaction; IC_50_ and Ki values were measured in proteoliposomes after the reconstitution procedure.

### 4.1. First Level Targets: Plasma Membrane Transporters

#### 4.1.1. The Glutamine/Neutral Amino Acid Transporters B0AT1 and ASCT2

The glutamine/neutral amino acid transporters B0AT1 and ASCT2 extracted from rat kidney and reconstituted in proteoliposomes have been found to be sensitive to mercurial compounds and some other heavy metal cations. HgCl_2_ and methyl-Hg were very effective in inhibiting the transport function of the two transporters. The compounds represent the main inorganic and organic forms of mercury, respectively, which contaminate the environment as byproducts of industries [108]. Mersalyl has been also tested since this compound is a hydrophilic mercury prototype, whose properties are well known being useful for comparison with the toxic compounds [109]. In the case of ASCT2, also Cu^2+^ which is known for its toxicity, exerts effects blocking the transporter function [110] (Table 2), while other heavy metals had slight or no effects on the amino acid transporters. The basis for the interaction of mercurials and Cu^2+^ consists in the known reactivity of these compounds towards thiol groups, which are present as Cys residues in the transporters. Very interestingly, the Cys residues in higher eukaryotic transport systems are more numerous than in transporters, and generally in proteins, of lower living organism [111]. This feature makes the mammalian transporters more sensitive to toxicity by heavy metals as in the described cases. The presence of Cys residues, that can be easily deduced from the transporter sequence, is the prerequisite for testing such type of compounds. Interestingly both the transporters contain a CXXC motif which is typical of metal binding sites in proteins. B0AT1 contains, in addition, a CXXXC motif which has been described previously as target of metals such as Cu^2+^, Pb^2+^, Hg^2+^, Cd^2+^ and Zn^2+^ [104,112]. However, only Hg^2+^ and, on ASCT2, Cu^2+^ exerted effects on these transporters, indicating that the interaction is strongly influenced by amino acid residues surrounding the target Cys, which are obviously very different in the two transporters. Another important requisite is that one or more Cys residues should be located in a site of the protein structure which is reachable by the reagents added to the extraliposomal (extracellular) compartment. The investigated transporters possess both the requisites, since the mercurials exerted effects on the function of both of them [91,93,110] at reasonably low concentrations. According to the working plan of Figure 3, additional analyses have been performed. The dose-response studies revealed that the mercury compounds are potent inhibitors of the two transporters (Table 2). In particular, both transporters had a lower affinity for mersalyl respect to the other mercurials. In general, ASCT2 revealed a lower affinity for all mercurials respect to B0AT1. As a proof of the interaction of the mercury compounds with thiol groups, the reducing agent DTE was tested for its capacity to reverse the inhibition (binding). The positive response to this test, confirmed the covalent reactions of the mercurials with the thiol group of Cys residue(s). More interestingly, endogenous and/or exogenous non toxic antioxidants such as L-cysteine and NAC also reversed the inhibition. Some differences in the effect of the antioxidants were found between the two transporters: the inhibition by mercurials was fully reversed in the case of ASCT2 but not in the case of B0AT1. This may correlate with the affinity of the B0AT1 for the mercury compounds (Table 2), which renders the reverse of the covalent bond more difficult. The scavenger ability of the antioxidants might be of interest for potential use in mercury detoxification. According to the working plan of Figure 3, additional information on the mechanisms was obtained by kinetic analysis of the inhibition. In all cases a non-competitive mechanism of inhibition was found both on glutamine and Na^+^ transport. However, in case of non competitive inhibition given by covalent bonds, as in the case of these reagents, it cannot be excluded that the reagents interact with residues located in the substrate binding site, therefore, further analyses are required. An efficient experimental trick to clarify this point is the test of substrate protection, *i.e.*, the effect of the presence of substrate on the inhibition. If the substrate prevents the inhibition, then the Cys responsible for the interaction is located close to the substrate binding site; if, on the contrary, the substrate has no effect on the inhibition, then the location of the Cys residue is confirmed to be far from the substrate binding site [110]. In both cases, the absence of protection confirmed that the Cys residues responsible for the inhibition are located far from the substrate binding site. However, in the case of B0AT1, protection towards the prototypal reagent mersalyl was found, indicating the presence of an additional site of interaction with mercurials, close to the substrate binding site, to which only mersalyl could bind. The results fit very well with the homology structural models of B0AT1 and ASCT2, built respectively on the templates LeuT and Glt_Ph_ [93,110]. Indeed, the CXXC motifs are located far from the hypothetical substrate binding site in agreement with the non competitive inhibition. The second motif CXXXC of B0AT1, in agreement with the data on mersalyl, is located close to the substrate binding site [91,93].

**Table 2:** IC_50_ values and half saturation constants of the reconstituted plasma membrane transporters ASCT2, Alanine, Serine, Cysteine, Transporter 2 (ASCT2), Broad specificity Amino acid Transporter 1 (B0AT1) and Organic Cation Transporter Novel 2 (OCTN2).

Molecules	ASCT2		B0AT1		OCTN2		References
	IC_50_	Ki	IC_50_	Ki	IC_50_	Ki	
HgCl_2_	1.4	0.85	0.42		2.5	4.2	[93,110,113]
MethylHg	2.4	1.1	0.89	0.33	7.4	13	[93,110,113]
Mersalyl	3.1	3.3	2				[91,110]
Cu^2+^	20						[110]
1,2,3,-dithiazoles	3–30						[49]
Omeprazole ^(^*^)^					5.7	5.2	[114]
Omeprazole ^(^**^)^					20.4	14.6	[114]

IC_50_ and Ki values are reported as µM; ^(^*^)^ covalent interaction ^(^**^)^ non covalent interaction; IC_50_ and Ki values were measured in proteoliposomes after the reconstitution procedure.

An important finding concerning the ASCT2 transporter is its up-regulation in several tumors. These cells use carbon atoms of glutamine and glucose as energy fuel instead of lipids. The altered expression profile of this protein was postulated to be important in tumor cells for net import of high amounts of glutamine for energy purposes, thus being a potential target for antitumor therapy. In this respect, proteoliposome studies gave important information on the actual role of ASCT2 in tumors. Indeed, as it was described for the rat transporter [90] and very recently for the human one [96], ASCT2 catalyzes an asymmetric antiport of amino acids. Thus the transporter cannot mediate net uptake of glutamine, but can only take up the amino acid in exchange with other smaller amino acids such as serine which has a lower free energy content. The rat ASCT2 in proteoliposomes was assayed for inhibition by 1,2,3-dithiazoles, which interact with thiol groups as potential antitumor agents [49]. Interestingly, some of these compounds are potent inhibitors of ASCT2. As for the mercury reagents, the DTE test demonstrated that also in this case the interaction is mediated by thiol groups of Cys. Since few months proteoliposomes with the human ASCT2 transporter are available for testing similar compounds on the human transporter.

#### 4.1.2. The Carnitine Transporter OCTN2

Organic cation transporters constitute a large group of transporters which are involved in interactions with drugs. Some of these transport systems, such as OCT1 and OCT2 [115] do not play specific physiological functions but have been found to be involved in the transport of several drugs thus, being important for drug delivery. The Package Inserts (PIs) of recently approved molecules, indeed, mention these proteins [54]. The SLC22A family, besides OCTs and OATs, comprises another sub-family which, differently from the OCTs, has important functions for cell homeostasis. This aspect has been extensively dealt with in a number of reviews [42,59] suggesting that these transporters are potential target of xenobiotics and some drugs. In this scenario, proteoliposomes revealed as a powerful tool to specifically investigate reaction mechanisms underlining OCTN interactions with environmental pollutant, drugs as well as substrate derivatives.

The homology structural models for these proteins are not available, since no bacterial counterparts have been crystallized so far. A model has been put forward for OCTN1, which however lacks the 142 amino acid large extracellular loop, thus being unsuitable for molecular interaction analysis [116]. The only available tool for molecular prediction in this case is the hydropathy profile of OCTN subfamily members, which shows that these proteins share some sequence features, such as the presence of seven Cys residues [30,59]. According to this model, four Cys residues are exposed towards the extracellular side of the transporter; whereas the others are located in the transmembrane segments. This topology suggests that SH groups are likely to be targeted by exogenous/endogenous compounds. In order to evaluate the importance of SH groups in the interactions with xenobiotics the best-known member of OCTN subfamily, OCTN2 has been used as model. Using the proteoliposome experimental model [89], the interaction of rat OCTN2 with mercurial agents HgCl_2_ and methyl-Hg has been assessed. The studies demonstrated that these toxic environmental pollutants, as for the glutamine transporters ASCT2 and B0AT1, strongly inhibited the carnitine transport mediated by OCTN2 by interaction with Cys thiols. Interestingly, the mechanisms observed quite well overlapped those found for the amino acid transporters. Inhibition indeed, was reversed by DTE as well as by L-cysteine, and NAC even though at different extent. From kinetic analysis the IC_50_ and Ki for HgCl_2_, and methyl-Hg were calculated to be in the micromolar range (Table 2), with a non competitive and mixed mechanism of inhibition, respectively. Similarly to the interaction between B0AT1 and mersalyl, the presence of the physiological substrate, *i.e.*, carnitine, prevented the inhibition caused by both HgCl_2_ and methyl-Hg indicating that the deputed thiol groups are located in the active site of the transporter. Differently from the amino acid transporters the tested mercurials agents could be also transported by OCTN2 specifically in antiport with intraliposomal carnitine [113]. Besides pollutants, many drugs have been tested on OCTN2, among which omeprazole exerted an effect on the transporter which was compatible with the work plan of Figure 3. Omeprazole is largely employed as proton pump inhibitor; its molecular mechanism has been well described [117] and is based on mixed disulfides formation with Cys residues exposed in the gastric lumen. Given this chemical property, omeprazole has been added to the external compartment of proteoliposomes prepared with functional OCTN2 extracted from rat kidney; in this experimental setting omeprazole revealed to be able in strongly reducing carnitine transport [114]. Differently from the interaction with mercurials, the inhibition exerted by omeprazole was only partially reversed by DTE. From kinetic analysis it was revealed that omeprazole interacts with OCTN2 with an additional competitive (non-covalent) mechanism (Table 2). The non-covalent interaction occurring with the active site of the transporter is explained on the light of the structural features of omeprazole that satisfy the minimal requirements of a molecule to be a potential interactor, of OCTN2 [89,114]. An intriguing finding was that the presence of the physiological substrate carnitine, did not protect but stimulated the inhibition. This is explained by the occurrence of conformational changes in the OCTN2 active site which could render the transporter more prone to reaction with omeprazole. The results obtained with mercurials and omeprazole can have important outcomes both in basic and in applied research. The side effects of omeprazole mimic, in a milder form, the clinical manifestations of primary carnitine deficiency, a genetic disease caused by mutations in the gene coding for OCTN2; at the same time the toxicity exerted by mercurials can be explained as alteration of cell homeostasis due to impaired transport of nutrients and cofactors or to altered redox sensing function of OCTN2. The described observations are confirmed by the fact that plasmatic concentration of omeprazole and mercurials is in the range of the measured Ki values (Table 2).

The plasma membrane transporter OCTN2 in proteoliposomes allowed also the evaluation of the interaction with carnitine derivatives, aimed to find molecules that can be easily absorbed by means of the transporter function. In this scenario the attention has been focused on NO-donor drugs since diffuse and severe pathologies, such as ischemia, respond to NO. The availability of NO-donor drugs can improve NO bioavailability. With this aim some carnitine nitro-derivatives molecules were designed and the capacity of OCTN2 to transport these compounds was evaluated in proteoliposomes [118].

### 4.2. Second Level Targets: Mitochondrial Transporters

The interaction of xenobiotic molecules has been also evaluated on the mitochondrial carnitine/acylcarnitine transporter (CACT) and ornithine/citrulline transporter (ORCT). The two transporters mainly catalyze antiport (Figure 2) of substrates across the inner mitochondrial membrane [119,120]. The CACT has an essential role in fatty acid β-oxidation. It mediates the transport of fatty acyl units as acylcarnitines into the mitochondrial matrix, where the enzymes of the β-oxidation pathway are located. Alterations of the CACT function will impair the β-oxidation and hence the energetic metabolism, as it has been demonstrated by the occurrence of the secondary carnitine deficiency [121]. The ORCT is an ornithine/citrulline antiporter, which is essential for the urea cycle in liver. It mediates entry of ornithine into the mitochondrial matrix and efflux of citrulline to connect the cytosolic and mitochondrial phases of the urea cycle. Also, in this case, genetic alterations have been found which cause the HHH-syndrome [122], demonstrating the essential function of this transporter.

#### 4.2.1. The Carnitine/Acylcarnitine Transporter, CACT

Among many pharmacological largely diffused compounds tested, some β-lactam antibiotics led to positive tests of inhibition on the CACT [106]. Indeed, in proteoliposomes, it has been possible to measure a specific inhibition of the transport activity of CACT by these drugs. After testing the effect of the antibiotics according to the work plan of Figure 3, the dose-response of each antibiotic was studied. It has to be noted that in this case, much higher IC_50_ values were considered to be significant (Table 1). This is due to the corresponding higher doses of administration of these antibiotics with respect to other pharmacological compounds. Inhibition kinetics performed with cefonicid and ampicillin, indicated a reversible competitive type of inhibition. This mechanism is not significant *in vivo*, because the related IC_50_ are much higher than the concentrations of these antibiotics in human plasma. However, cefonicid can form a covalent and irreversible bond with the protein when incubated with proteoliposomes for 60 h. Under this condition much lower IC_50_, close to the plasmatic concentrations of cefonicid were measured. Thus, significant effect may be evident *in vivo* after long time administration of these drugs, with severe consequences on the mitochondrial β-oxidation of fatty acids and then in the production of metabolic energy. Accordingly, it was observed that some side effects of this antibiotic were similar to the symptoms described for the secondary carnitine deficiency, caused by genetic defects of CACT.

Another drug that affects the function of CACT is mildronate [3-(2,2,2-trimethylhydrazine) propionate]. This compound displays a chemical structure very similar to carnitine. It strongly inhibited the transport activity of CACT when externally added to the proteoliposomes together with the physiological substrate. Also in this case, according to the work plan of Figure 3, the dose-response and kinetic analysis were performed (Table 1). A pure competitive inhibition of carnitine transport by mildronate was observed, indicating an interaction with the substrate binding site. This correlated well with the structure similarity of mildronate and carnitine. It was also demonstrated that mildronate can be transported in antiport with carnitine. These findings allowed us to predict a potential mechanism at the basis of the administration of mildronate in pharmacological therapy explaining the cardiomyopathy or the liver steatosis observed in rats after a long treatment. Mildronate can influence *in vivo* the β-oxidation of fatty acids, hindering the uptake of acylcarnitines and depleting the intramitochondrial pool of carnitine, which is exchanged with cytosolic mildronate [107].

The interaction of the CACT with H_2_O_2_ has been revealed [105]. H_2_O_2_ is formed in cells, mostly in mitochondria, playing signaling roles; its concentration can increase reaching local millimolar range under pathological conditions [123,124]. The inhibitory action by H_2_O_2_ at physiological concentrations, *i.e.*, less than 50 μM, is about 30%. The inhibition became much more effective at higher concentration of the reactant (Table 1). Therefore, the transporter can be blocked only under pathological conditions such as in the presence of oxidative stress. Since CACT is an essential player of the fatty acid β-oxidation pathway, on the basis of the results described it can be assumed that the metabolic pathway can be finely tuned by physiological concentrations of H_2_O_2_; while under pathological conditions, the increase in H_2_O_2_ concentration can severely impair β-oxidation. The kinetic analysis highlighted that the inhibition exerted by H_2_O_2_ is of the competitive type and, accordingly, it was also observed that the presence of substrates of the transporter such as carnitine or acylcarnitines prevents CACT inhibition. It was also demonstrated by reversing the inhibition with DTE, that H_2_O_2_ interacts with Cys residues. Owing to the availability of the recombinant protein, site-directed mutagenesis of CACT could be performed on Cys residues. The six Cys were substituted with serine or valine constructing single and multiple mutants, lacking one or more of the Cys residues. This experimental strategy allowed to identify the Cys residues oxidized in the presence of H_2_O_2_. It has been demonstrated that H_2_O_2_ completely inactivates the transporter by inducing the formation of a disulphide between Cys-136 and Cys-155 [105]. NAC and L-cysteine removed the inhibition caused by H_2_O_2_.

#### 4.2.2. The Ornithine/Citrulline Transporter, ORCT

Effects of agents ranging from pharmaceutical and toxic compounds to physiological effectors have been tested on the mitochondrial ORCT which is highly expressed in liver where it is essential for the functioning of the urea cycle [122,125,126]. Different effects of sulfhydryl reagents have been evidenced on this transport protein. On the one hand, the inhibition of the transport function by metanthyosulphonates, *N*-ethylmaleimmide and disulphide forming compounds was evident; on the other hand, the induction of a pore like activity caused by higher (micromolar) pOHMB was observed [102]. The transition from the physiological to the unphysiological pore like unidirectional transport function was previously described for other mitochondrial transporters as induced by modification of Cys residues [102] and it has been proposed that this switch should be related to altered mitochondrial permeability. In the reconstituted system it has been demonstrated that the “concentration dependent” behavior of mercurials is related to a cysteine population different from the one responsible of the inhibition. These studies performed with prototypal chemical reagents, served as model for understanding the behavior of the transporter. Then, the effect of potentially toxic heavy metal cations on the ORCT has been highlighted in proteoliposomes. The cations dealt with are normally present in human blood at concentrations depending on the cation and the environmental conditions. Their concentration can increase due to exposition to pollutants. Accurate measurement of the IC_50_ and Ki values for each metal cation has been performed (Table 1) allowing the prediction of toxic effect related to the transport protein. Prediction of liver toxicity has been postulated in these studies.

## 5. Conclusions

Proteoliposomes have been used since long time, for studying membrane transporters in isolated environment. In the last decade, improved methodologies allowed to study kinetics and transport mechanisms of several plasma membrane transporters. More interestingly, the summarized novel results, highlight the power of proteoliposomes in revealing the molecular mechanisms of the interactions of transporters with different types of xenobiotics, from toxic molecules to commonly used drugs or newly synthesized compounds. In particular, aspects of “off-site” interactions have been revealed, which may be important in predicting side effects and, hence, in improving drug design. Bioinformatic analyses correlated well with the experimental data thus confirming the suitability of proteoliposomes for obtaining information on molecular mechanisms. Therefore, proteoliposomes can be considered a new tool, among those already available, for studying the role of transporters in drug development and clinical pharmacology [39].

## Abbreviations

OCTN: Organic Cation Transporter Novel
ASCT2: Alanine, Serine, Cysteine, Transporter 2
B0AT1: Broad specificity Amino acid Transporter 1
TEA: tetraethylamonium
CACT: Carnitine/Acyl-Carnitine Transporter
ORCT: Ornithine/Citrulline transporter
LeuT: Leucine Transporter
Glt_Ph_: Glutamate transporter from *Pyrococcus horikoshii*
methyl-Hg: methyl mercury
NAC: *N*-Acetyl Cysteine
